# CircMAN1A2 is upregulated by *Helicobacter pylori* and promotes development of gastric cancer

**DOI:** 10.1038/s41419-022-04811-y

**Published:** 2022-04-28

**Authors:** Ruiting Guo, Xixi Cui, Xue Li, Wen Zang, Mingjie Chang, Zenghui Sun, Zhifang Liu, Yundong Sun, Jihui Jia, Wenjuan Li

**Affiliations:** 1 Key Laboratory for Experimental Teratology of Chinese Ministry of Education, The Shandong Provincial Key Laboratory of Infection and Immunology, Department of Pathogenic biology, School of basic medical sciences, Shandong University-Karolinska Institutet Collaborative Laboratory for Cancer Research, Shandong University, Jinan, PR China; 2 Rizhao Maternal and Child Health Care Hospital, Rizhao, PR China; 3 Department of Biochemistry and Molecular Biology, School of basic medical sciences, Shandong University, Jinan, PR China

**Keywords:** Cancer, Non-coding RNAs

## Abstract

*Helicobacter pylori* (*H. pylori*) is one of the main causes of gastric cancer. It has been reported that circRNAs play a vital role in the development of multiple types of cancer. However, the role of *H. pylori*-induced circRNAs in the development of gastric cancer has not been studied. In this study, we found that *H. pylori* could induce the upregulation of circMAN1A2 in AGS and BGC823 cells independent of CagA. The downregulation of circMAN1A2 could inhibit the proliferation, migration and invasion of gastric cancer cells, and circMAN1A2 could promote the progression of gastric cancer induced by *H. pylori* by sponging miR-1236-3p to regulate MTA2 expression. Furthermore, circMAN1A2 knockdown inhibited xenograft tumour growth in vivo, and the overexpression of circMAN1A2 was associated with the progression of gastric cancer. Hence, *Helicobacter pylori* induced circMAN1A2 expression to promote the carcinogenesis of gastric cancer, and circMAN1A2 might be a new potential diagnostic marker and therapeutic target for gastric cancer.

## Introduction

Gastric cancer (GC) is one of the most common malignant tumours in the world. According to statistics in 2018, the incidence and mortality of gastric cancer in East Asia ranks first in the world [1, 2]. Once they are diagnosed, most patients are in an advanced stage of GC with poor prognosis [3], and thus, it is extremely important to study the pathogenesis of GC. The occurrence of GC is a multifactor and multilevel process. It has been demonstrated that *Helicobacter pylori* (*H. pylori*) is the main environmental factor that can induce GC, and *H. pylori* infection is an important initial factor for the development of GC [4–6]. Studies have also shown that *H. pylori* can give rise to cancerization by regulating the expression of noncoding RNA to induce proliferation and apoptosis of gastric epithelial cells [7–9].

With the development of high-throughput sequencing technology, circRNA, which has a highly conserved and stable covalently closed cyclic structure, has aroused interest [10]. CircRNA is abundant and stable in eukaryotic cells, and it can survive under RNase R treatment [11, 12]. CircRNA was originally thought to be a by-product of mRNA production during mis-splicing without biological functions [13, 14]. However, thousands of circRNAs have been found to be involved in the regulation of gene expression and play a significant role in cell differentiation, tissue homeostasis, and disease development [15–17]. It is thought to be one of the most important biological functions that circRNAs can act as miRNA sponges to improve the expression level of miRNA target genes by inhibiting the activity of miRNAs [18, 19]. For example, circHIPK3 can sponge multiple miRNAs to regulate cell growth; [20] circIRAK3 acts as a miR-3607 sponge to promote breast cancer metastasis; [21] and circHLA-C can sponge miR150 to regulate the development of lupus nephritis [22]. Recent research reports that circRNAs are involved in the development of GC, but the mechanism of action of circRNAs in the development of GC is not fully understood [19]. *H. pylori* infection is an important factor in the malignant transformation of gastric mucosa. Studies have also shown that *H. pylori* can regulate the development of gastric cancer by regulating the expression of a variety of noncoding RNAs or mRNAs [23, 24]. However, there is no report about the role of circRNAs in GC induced by *H. pylori*.

Therefore, our study analysed the expression profile of circRNAs in gastric cancer cells infected by *H. pylori* by RNA sequencing. Then, circMAN1A2 with the most significant expression difference was selected according to the sequencing results to study the epigenetic mechanism of *H. pylori*-induced gastric carcinogenesis. We studied the role of circMAN1A2 in the development of gastric cancer induced by *H. pylori* and revealed the molecular mechanism by which circMAN1A2 promotes gastric mucosal malignant transformation, providing a basis for the prevention and early diagnosis of GC.

## Methods

### Cell lines, *H. pylori* strains and the infection model

GES-1 human gastric epithelial cells and AGS and BGC823 human gastric cancer cell lines and two *H. pylori* strains were cultured according to the methods described in our previous article [25].

### RNA-seq data analysis and identification

Pair readings were collected by the Illumina HiSeq Sequencer. The total number of readings in the obtained sample was used for normalization and log2 conversion, and the number of standardized readings was compared between the samples. The original reading and human reference genome (hg19) were compared via STAR software, and the Ensemble (V70) GTF file was used as a guide. To align high-quality reads with the reference genome/transcriptome, Bowtie2 software was used, and the find_circ software was used to detect and identify circRNAs. CircRNAs showing a fold-change > 2.0 and *P* value < 0.05 were classified as significantly differentially expressed circRNAs. Hierarchical clustering was performed using Heatmap2 in R software for circulating RNAs with a fold variation greater than 2.0.

### Small interfering RNA (siRNA), plasmid and lentivirus transfection

The circMAN1A2 siRNAs and miR-1236-3p were designed and purchased from the Suzhou Ribo Life Science Co., Ltd. (Suzhou, China). According to the sequence across the back-splicing junction site of circMAN1A2, circMAN1A2 siRNAs were designed to specifically knock down the expression of circMAN1A2. The pcDNA3.1-CagA plasmid was kindly provided by Yongliang Zhu (Zhejiang University, China). pcDNA3.1-circMAN1A2-WT and pcDNA3.1-circMAN1A2-Mut were purchased from the Hanbio Biotechnology Co., Ltd. (Shanghai, China). The siRNA and plasmid were transfected with jetRPIME transfection reagent (Polyplus, Illkirch, France). Lentiviruses of sh-NC and sh-circMAN1A2 were purchased from the Shanghai GeneChem Co., Ltd. (Shanghai, China). Cells stably transfected with sh-NC and sh-circMAN1A2 lentiviruses were screened by puromycin. The sequences of siRNAs were as follows:

si-negative control (si-NC): 5ʹ-UUCUUCGAAACGUGUCACGUT-3ʹ;

si-circMAN1A2-1: 5ʹ-AUUUCCUCUCUUGACUUUCTT-3ʹ;

si-circMAN1A2-2: 5ʹ-UUUCUGCUCGAAUUUCCUCTT-3ʹ.

### Patients and specimens

Fifty-two *Hp−* human gastritis specimens and 47 *Hp*+ human gastritis specimens were collected from patients undergoing gastroscopy. A total of 101 pairs of GC tissues were obtained from patients with GC during surgery. Preoperative plasma was obtained from 49 GC gastric cancer patients. The infection of *Helicobacter pylori* in gastritis patients was detected via ^14^C urea breath tests, and gastritis tissues were used to detect *H. pylori* infection by rapid urease test. All patient tissues and plasma were obtained from Qilu Hospital at Shandong University. Plasma from 46 healthy people was donated by volunteers. Our research was approved by the Ethics Committee of Shandong University School of Medicine (Jinan, China) and conducted in accordance with the Declaration of Helsinki.

### RNA preparation and qRT–PCR

TRIzol (Invitrogen, Waltham, MA, USA) was used to extract total RNA from cells and tissues. The purity and quantity of total RNA were then detected by a NanoDrop Lite Spectrophotometer (Thermo Scientific, USA).

Nuclear and cytoplasmic RNAs were extracted using NE-PER Nuclear and Cytoplasmic Extraction Reagents (Thermo Scientific, USA) according to the manufacturer’s protocol.

The RNA was reverse transcribed into cDNA with a Prime Script RT Reagent Kit (Takara Bio Inc, Osaka, Japan). qRT–PCR was conducted using a SYBR Premix Ex Taq System (Takara) and a CFX96 Real-time PCR System (Bio–Rad, USA). All of the primers are listed in Supplementary Table 1.

### Western blot

The high efficiency RIPA lysis fluid and the protease inhibitor were prepared into a protein lysate in a ratio of 100:1 to extract the total cell protein. The purity and concentration of total protein were then detected by NanoDrop Lite Spectrophotometer (Thermo Scientific, USA). Proteins were separated by SDS-PAGE and then transferred to polyvinylidene difluoride membranes (Merck Millipore, Germany), blocked in 5% skim milk for 1 h, and then incubated with anti-CagA primary antibody (1:500, Santa, sc-28368, USA), GAPDH (1:5000, Abways, AB0036, China) at 4 °C overnight. Anti-mouse and anti-rabbit horseradish peroxidase-conjugated secondary antibodies were incubated for 1 h at room temperature and exposure using enhanced chemiluminescence (Millipore, USA).

### CCK-8, colony formation and transwell assays

CCK-8, colony formation and transwell assays methods are detailed in the methods described in our previous article [25].

### RNase R treatment

A RNeasy MinElute Cleanup Kit (Qiagen, USA) was used to purify the resulting RNA. Total RNA (2 μg) was incubated with or without 3 U/μg RNase R at 37 °C for 20 min. RNA expression was detected by qRT–PCR.

### Dual-luciferase reporter assay

The pcDNA3.1-circMAN1A2-WT, pcDNA3.1-circMAN1A2-Mut and miR-1236-3p mimics were cotransfected into GC cells. After 48 hours of transfection, the luciferase activities were quantified with a dual-luciferase reporter assay (Promega, USA) detected by Centro XS^3^ LB960 (Berthold Technologies, Germany).

### Animal experiments

Four-week-old female BALB/c nude mice were procured from Beijing Vital River Laboratory Animal Technology (Beijing, China). Nude mice were randomly divided into two groups with 6 mice in each group. BGC823 cells stably transfected with sh-circMAN1A2 and sh-NC lentivirus were resuspended, and 100 µl of cell suspension containing 1 × 10^6^ cells was injected into the subcutaneous axilla of nude mice. The length and width of the tumour were measured with a Vernier caliper once a week and recorded for 4 weeks. After 4 weeks, the tumour was removed and weighed. This research was approved and guided by the Ethics Committee of Shandong University School of Medicine (Jinan, China).

### Statistical analysis

Statistical analysis was conducted using SPSS v25.0 (IBM Corp.) and Graphpad 8.0. All data were presented as the mean ± SD of at least three biological replicates. ANOVA with an appropriate post-hoc correction was used for comparisons among multiple groups. Student’s *t* test was generally used to analyze the differences between two groups, but when the variances differed, the Mann–Whitney *U* test was used. *P* < 0.05 (two-tailed) was considered to indicate a statistically significant difference.

### Date deposition

The circRNA sequencing data used in this study has been uploaded to the Gene Expression Omnibus (GEO) with the accession code GSE183628.

## Results

### Differentially expressed circRNAs in AGS cells infected with *Hp26695* vs. control

**Fig. 1:**
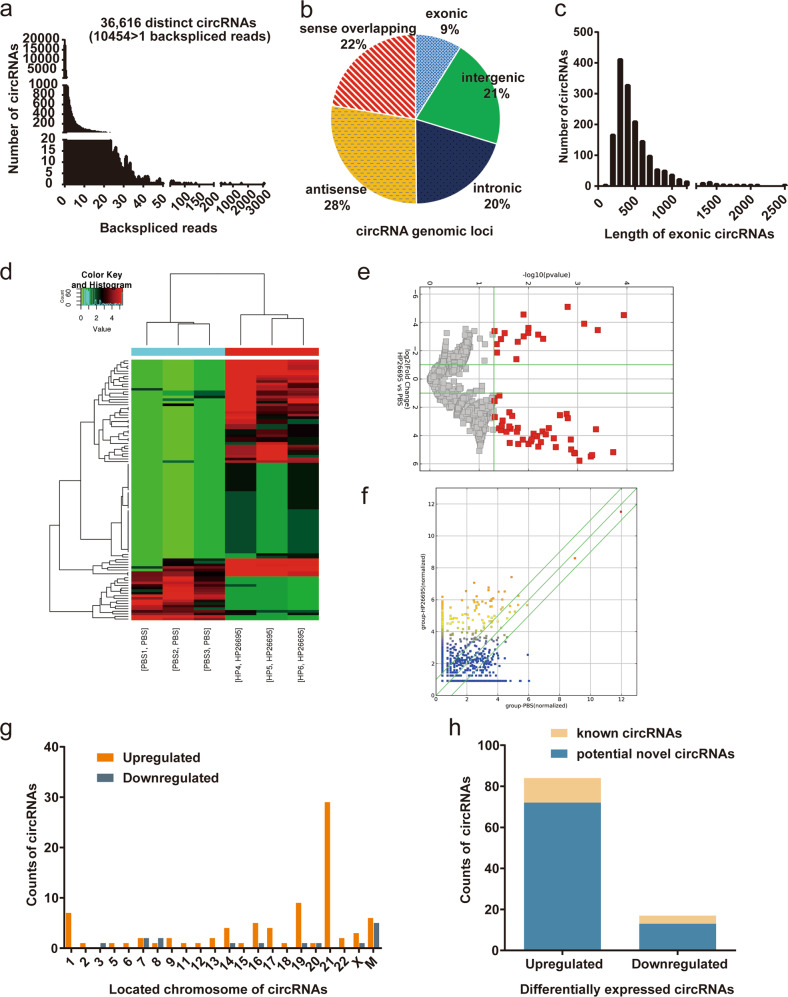
Differentially expressed circRNAs in AGS cells infected with *Hp26695* vs. control. **a** The number of reverse splicing events of circRNAs was identified in PBS-treated and *Hp*-infected AGS cells: 10,454 common circRNAs have two or more reverse splicing events. **b** The origin and distribution of circRNAs: exon circRNA (9%), intron circRNA (20%), intergenic region circRNA (21%), overlapping region circRNA (22%) and antisense circRNA (28%). **c** Length distribution of circRNAs (*n* = 18 308). **d** Heat map of the expression levels of circRNAs in AGS cells treated with PBS and *Hp26695*. (*n* = 6). Green to red represents circRNA expression from low to high, respectively. **e** Volcano plot of differentially expressed circRNAs: red dots indicate differentially expressed circRNAs; the left panel shows downregulated circRNAs, and the right panel shows upregulated circRNAs. (fold change > 2.0, *P* < 0.05, FDR < 0.05). **f** Scatter plots identified changes in circRNA expression (dots above the diagonal line indicate upregulated circRNAs, and dots below the diagonal line indicate downregulated circRNAs). **g** Chromosomal locus distribution of differentially expressed circRNAs. **h** The number of differentially expressed circRNAs.

To explore whether *H. pylori* infection could cause circRNA changes in GC, we treated AGS with PBS and *H. pylori* to perform RNA-seq analysis. A total of 18 308 different circRNA candidates were obtained in the experiment, of which 10 454 of these circRNAs contained at least two unique back-junction readings (Fig. 1a). Of these 18,308 circRNA candidates, only 1628 (8.9%) were exon-loop RNAs produced by the exon of the mononuclear protein-coding gene (Fig. 1b). The majority of circRNAs produced by the exon of the mononuclear protein-coding gene were less than 1500 nucleotides in length and had a median length of 500 (Fig. 1c).

Compared with the control, 101 significantly differentially expressed circRNAs were identified in the AGS cells infected with *H. pylori*, including 84 upregulated circRNAs and 17 downregulated circRNAs (Fig. 1d–f). The 101 differentially expressed circRNAs were located on all human chromosomes, including 22 autosomes, the X chromosome, and the mitochondria (Fig. 1g). Among the 84 upregulated circRNAs, 12 circRNAs were identified previously and listed in the published circRNA database or articles, and 72 circRNAs might be potential novel circRNAs that were identified for the first time. Among the 17 downregulated circRNAs, 4 circRNAs were identified previously, and 13 circRNAs might be potential novel circRNAs (Fig. 1h).

### *H. pylori* could upregulate the expression of circMAN1A2 in gastric cancer cells

**Fig. 2:**
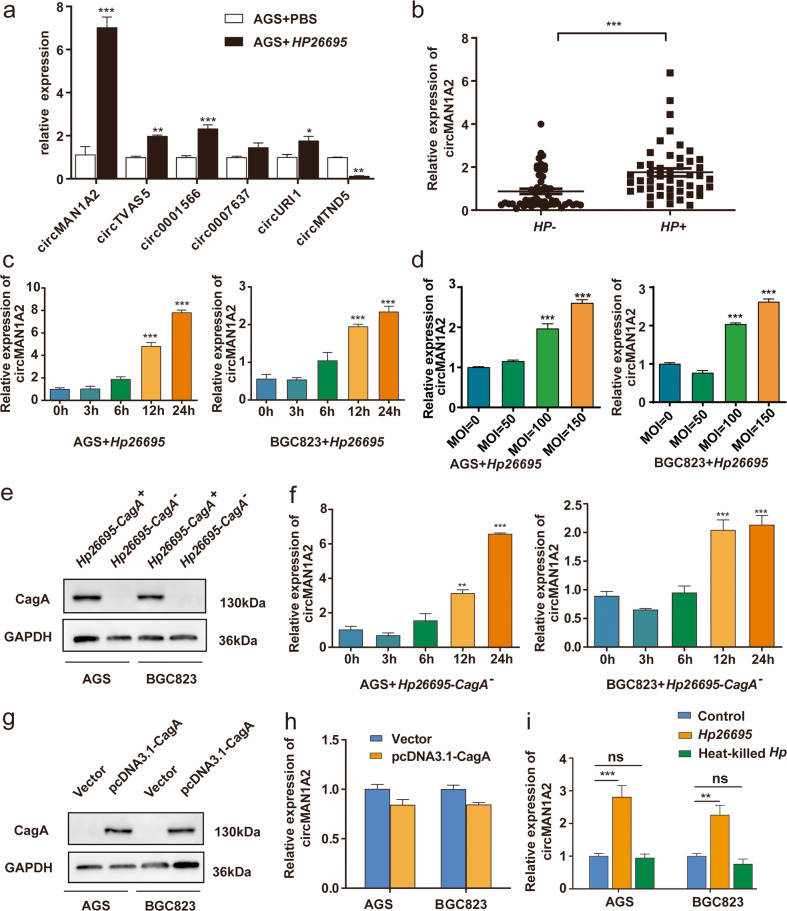
*H. pylori* upregulated the expression of circMAN1A2 in gastric cancer cells. **a** After *Hp26695*-infected AGS cells were infected at an MOI = 100:1 for 12 h, the expression of circRNAs was detected by qRT–PCR. The control group used PBS for the same amount of time. **b** qRT–PCR analysis of circMAN1A2 expression in *Hp*^+^ gastritis tissues (*n* = 47) and *Hp*^−^ gastritis tissues (*n* = 52). (Mann–Whitney U). **c** After *Hp26695* infected AGS and BGC823 cells at a MOI = 100:1 for different times (0, 3, 6, 12 and 24 h), the relative expression of circMAN1A2 was assessed by qRT–PCR. **d**. After *Hp26695* infected AGS and BGC823 cells with different MOIs (0, 50, 100, and 150) for 12 h, the expression of circMAN1A2 was detected by qRT–PCR. **e** Western blot was used to detect the expression of CagA protein in AGS and BGC823 cells after *Hp*26695-CagA^+^ and *Hp*26695-CagA^-^ infection for 24 h. **f** After *Hp26695*-CagA^-^ infected AGS and BGC823 cells at a MOI = 100:1 for different times (0, 3, 6, 12 and 24 h), the expression of circMAN1A2 was detected by qRT–PCR. **g** CagA protein in AGS and BGC823 cells transfected with the PcDNA3.1-CagA plasmid and PcDNA3.1 plasmid was analysed by Western blot. **h** qRT–PCR detected the expression of circMAN1A2 in AGS and BGC823 cells transfected with the PcDNA3.1-CagA plasmid and PcDNA3.1 plasmid. **i** After *Hp26695* and heat-killed *Hp26695* infected AGS and BGC823 cells at a MOI = 100:1 for 12 h, the expression of circMAN1A2 was assessed by qRT–PCR. The control group used PBS. *P < 0.05, **P < 0.01, ***P < 0.001.

Five upregulated circRNAs and one downregulated circRNA were selected based on the difference in gene expression, and the expression levels of these circRNAs were verified in AGS cells infected with *H. pylori*. The changes in circRNA expression detected by qRT–PCR were consistent with the RNA-seq data (Fig. 2a). Since the expression difference of circMAN1A2 was the most significant, we chose circMAN1A2 for further verification. We also identified that the expression of circMAN1A2 was much higher in *Hp*^*+*^ chronic gastritis tissues than in *Hp*^*−*^ chronic gastritis tissues (Fig. 2b). Then, we infected AGS and BGC823 cells with *Hp26695* at a specific time and concentration. The results showed that *Hp26695* could significantly upregulate the expression of circMAN1A2 when it infected AGS and BGC823 cells for 12 and 24 h, or at the concentration of MOI = 100 and 150 (Fig. 2c, d).

CagA is one of the most important virulence factors of *H. pylori*, and it can affect multiple pathways in cells. Therefore, we speculated whether the upregulation of circMAN1A2 depended on CagA. We detected the expression of CagA protein in AGS and BGC823 cells after *Hp26695*-CagA^+^ and *Hp26695*-CagA^−^ infection for 24 h (Fig. 2e). However, we found that circMAN1A2 was also overexpressed in AGS and BGC823 cells infected with *Hp26695* with CagA depletion for 12 and 24 h. (Fig. 2f). Then we detected the expression of cagA plasmid in gastric cancer cells and found that the CagA plasmid could not upregulate the expression of circMAN1A2 (Fig. 2g, h). Meanwhile, heat-killed *Hp26695* did not upregulate the expression of circMAN1A2 (Fig. 2i), indicating that the upregulation of circMAN1A2 induced by *H. pylori* did not depend on CagA but might depend on the interaction between living bacteria and cells.

### CircMAN1A2 could promote the proliferation, migration and invasion of gastric cancer cells

**Fig. 3:**
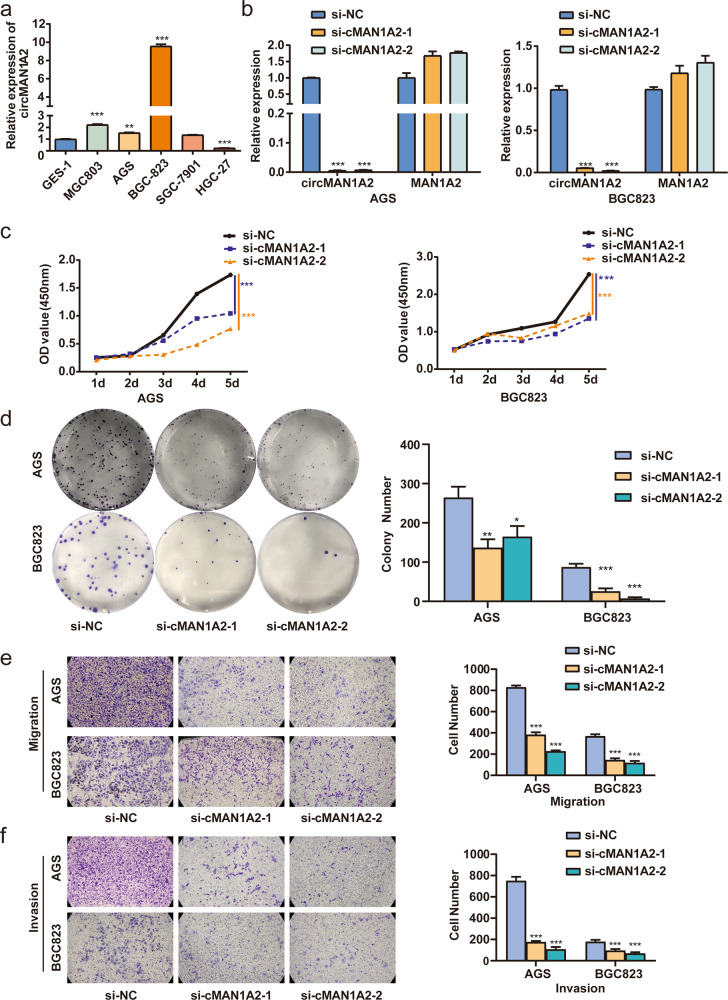
CircMAN1A2 could promote the proliferation, migration and invasion of gastric cancer cells. **a** qRT–PCR analysis of circMAN1A2 in the human immortalized gastric epithelial cell line GES-1 and gastric cancer cell lines MGC803, AGS, BGC823, SGC7901 and HGC27. **b** The interference efficiency of circMAN1A2 siRNAs was detected by qRT–PCR in AGS cells and BGC823 cells. **c** The proliferation ability of AGS and BGC823 cells transfected with circMAN1A2 siRNAs was detected by CCK-8 assay at 5 time points (24, 48, 72, 96 and 120 h). **d** The colony formation test was used to detect the clonogenic ability of AGS and BGC823 cells transfected with circMAN1A2 siRNAs. **e** The migration ability of AGS and BGC823 cells transfected with circMAN1A2 siRNAs was detected by Transwell assays without Matrigel. **f** The invasion ability of AGS and BGC823 cells transfected with circMAN1A2 siRNAs was measured by Transwell assays with Matrigel. *P < 0.05, **P < 0.01, ***P < 0.001.

Then, we selected AGS and BGC823 cells with higher expression levels of circMAN1A2 for functional verification in vitro (Fig. 3a). We first knocked down the expression of circMAN1A2 in AGS and BGC823 cells with siRNA (Fig. 3b). The CCK-8 and colony formation results demonstrated that the downregulation of circMAN1A2 could inhibit the proliferation of GC cells (Fig. 3c, d). Transwell assays without or with Matrigel showed that the downregulation of circMAN1A2 could inhibit the migration and invasion of GC cells (Fig. 3e, f).

### CircMAN1A2 could act as a miR-1236-3p sponge in GC cells

**Fig. 4:**
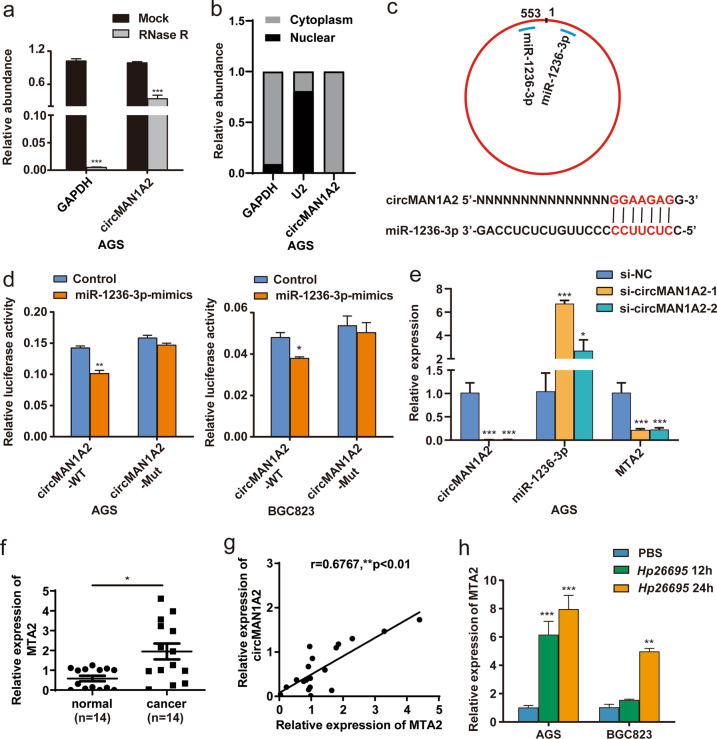
CircMAN1A2 could act as a miR-1236-3p sponge in GC cells. **a** After RNase R treatment, the tolerance of circMAN1A2 to RNase R was detected by qRT–PCR. **b** qRT–PCR was used to detect the expression of circMAN1A2 in the nucleus and cytoplasm. GAPDH was used as the cytoplasmic internal reference, and U2 was used as the nuclear internal reference. **c** Schematic diagram of the binding site of circMAN1A2 and miR-1236-3p and the binding sequence of circMAN1A2 and miR-1236–3p. **d** Luc-circMAN1A2-WT and Luc-circMAN1A2-Mut cotransfected AGS and BGC823 cells with miR-1236-3p mimics, respectively. The luciferase activity was detected by the dual-luciferase assay in AGS and BGC823 cells. **e** The expression of miR-1236-3p and MTA2 was detected by qRT–PCR after knockdown of circMAN1A2 in AGS and BGC823 cells. **f** The expression of MTA2 in normal adjacent tissues (*n* = 14) and gastric cancer tissues (*n* = 14) was detected by qRT–PCR. (Mann–Whitney U). **g** Spearman’s correlation coefficient analysis was used to assess the correlation between circMAN1A2 and MTA2 levels in GC tissues (*n* = 20). **h**. After *Hp26695* infected AGS and BGC823 cells at a MOI = 100:1 for different times (0, 12 and 24 h), the relative expression of MTA2 was assessed by qRT–PCR. *P < 0.05, **P < 0.01, ***P < 0.001.

To study how circMAN1A2 regulates the proliferation, invasion and migration of GC cells, we first confirmed the circular structure of circMAN1A2 by RNase R tolerance test (Fig. 4a). NE-PER nuclear and cytoplasmic extraction test showed that circMAN1A2 was mainly expressed in the cytoplasm (Fig. 4b). This suggested that circMAN1A2 might be involved in the function of gastric cancer cells by absorbing miRNA through sponging action. Therefore, we used RNAhybrid and miRanda software to predict the miRNAs that might bind to circMAN1A2 and found that miR-1236-3p had a stronger binding ability to circMAN1A2 (Fig. 4c). Then, we designed a luciferase reporter assay to further confirm this hypothesis. The luciferase signal decreased when Luc-circMAN1A2-WT was cotransfected with miR-1236-3p mimics into AGS cells. When Luc-circMAN1A2-Mut was cotransfected with miR-1236-3p mimics into AGS and BGC823 cells, no change in the luciferase signal was observed. This finding suggested that circMAN1A2 could bind to miR-1236-3p by the miRNA sponging effect (Fig. 4d).

The expression of miR-1236-3p was reduced in GC tissues, and its expression was related to lymph node metastasis, differentiation and the clinical stage of GC [26, 27]. The overexpression of miR-1236-3p could repress the proliferation, migration and invasion of GC cells by inhibiting MTA2, and the EMT and PI3K/AKT pathways could be suppressed by miR-1236-3p in GC cells [28–30]. We found that after knockdown of circMAN1A2, the expression of miR-1236-3p was upregulated, while the expression of MAT2 mRNA was decreased in GC cells (Fig. 4e). Then, we found that the expression of MTA2 was overexpressed in GC tissues and was positively correlated with circMAN1A2 in GC tissues (Fig. 4f, g). Moreover, the mRNA expression level of MTA2 was upregulated when induced by *H. pylori* in AGS and BGC823 cells (Fig. 4h). These results further indicated that circMAN1A2 could regulate the development of GC through the miR-1236–3p/MTA2 axis.

### *H. pylori* enhanced GC cell migration by upregulating the expression of circMAN1A2 in vitro

**Fig. 5:**
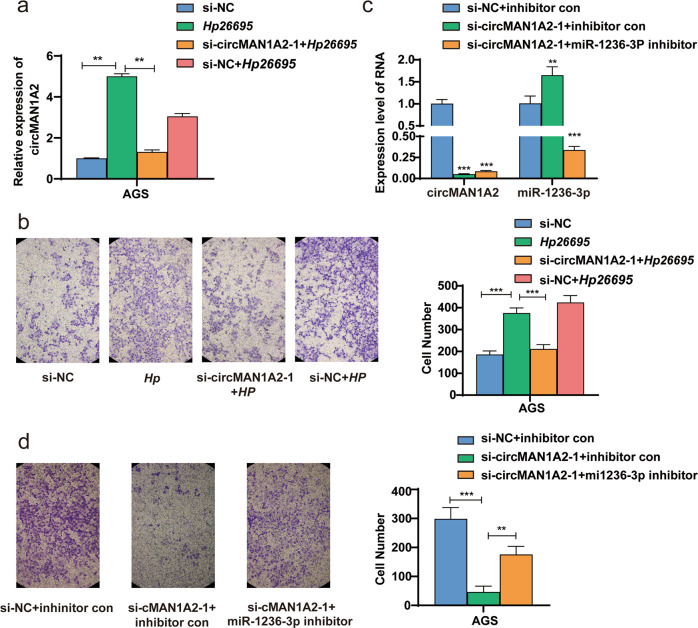
*H. pylori* promotes the migration of GC cells by regulating the circMAN1A2-miR-1236-3p axis. **a** The expression of circMAN1A2 after *H. pylori* infection and transfection of circMAN1A2 siRNAs in AGS cells was detected by qRT–PCR. **b** The migration ability of AGS cells after *H. pylori* infection and transfection of circMAN1A2 siRNAs was detected by Transwell assays without Matrigel. **c** The expression of circMAN1A2 and miR-1236-3p after transfection of circMAN1A2 siRNAs and miR-1236-3p inhibitor in AGS cells was detected by qRT–PCR. **d** The migration ability of AGS cells after transfection of circMAN1A2 siRNAs and miR-1236-3p inhibitor was detected by Transwell assays without Matrigel. *P < 0.05, **P < 0.01, ***P < 0.001.

We found that circMAN1A2 could promote the proliferation and migration of GC cells and *H. pylori* could promote the progression of gastric cancer. To verify whether the effect of *H. pylori* on gastric cancer depends on circMAN1A2, we infected AGS cells with *Hp26695*. Twelve hours later, si-circMAN1A2–1 was transfected into the infected cells. The relative expression of circMAN1A2 was detected with qRT–PCR (Fig. 5a). Through Transwell assays without Matrigel, we found that *H. pylori* enhanced the migration ability of AGS cells, which was weakened by circMAN1A2 siRNAs (Fig. 5b). This result showed that *H. pylori* could enhance GC cell migration by upregulating the expression of circMAN1A2 in vitro.

### CircMAN1A2 could promote the migration of AGS by regulating miR-1236-3p

According to the above experimental results, circMAN1A2 could act as a miR-1236-3p sponge in GC cells. Therefore, we designed a rescue experiment to determine whether the regulation of circMAN1A2 on gastric cancer progression depended on miR-1236-3p. We interfered with both circMAN1A2 and miR-1236-3p, and the expression of circMAN1A2 and miR-1236-3p was examined by qRT–PCR (Fig. 5c). The results of Transwell experiments without Matrigel showed that circMAN1A2 siRNAs could inhibit the migration ability of AGS cells, which was weakened by miR-1236-3p inhibitor (Fig. 5d). This indicated that circMAN1A2 could promote the migration of AGS by binding to miR-1236-3p.

### Downregulation of circMAN1A2 inhibits the proliferation of gastric cancer cells in vivo

**Fig. 6:**
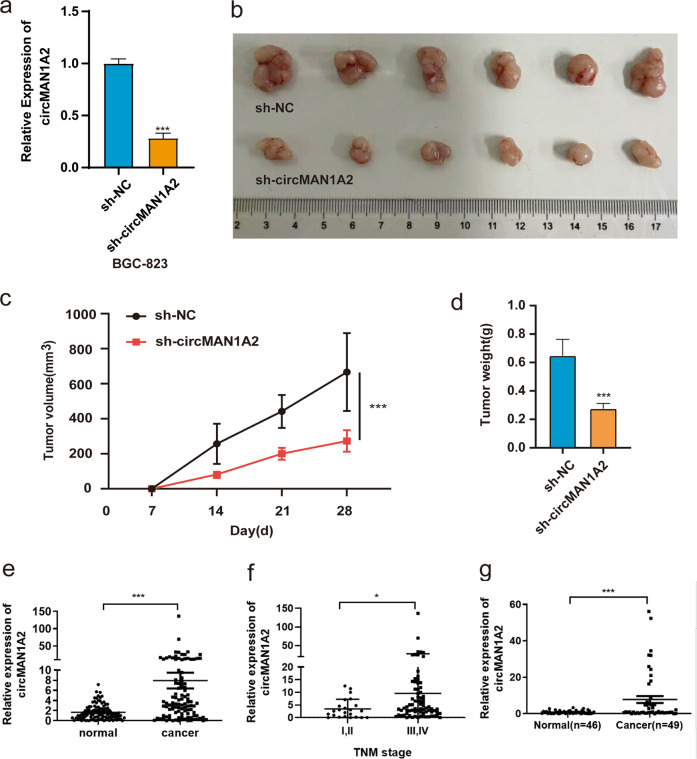
CircMAN1A2 knockdown inhibited xenograft tumour growth in vivo and the clinical relevance of circMAN1A2 in GC. **a** qRT–PCR analysis of circMAN1A2 expression in BGC823 cells transfected with sh-circMAN1A2 and sh-NC. **b** The tumours of the xenograft mouse model were removed and imaged. **c**, **d** The tumour volume and weight were measured and analysed. **e** The expression of circMAN1A2 in normal adjacent tissues (*n* = 101) and gastric cancer tissues (*n* = 101) was detected by qRT–PCR. **f** qRT–PCR analysis of circMAN1A2 expression in gastric cancer tissues in different TNM stages. **g** Relative expression of circMAN1A2 assessed by qRT–PCR in the plasma of gastric cancer patients (*n* = 49) and healthy donors (*n* = 46). *P < 0.05, **P < 0.01, ***P < 0.001.

Previous studies showed that downregulating the expression of circMAN1A2 can significantly inhibit the proliferation of gastric cancer cells in vitro. Therefore, we established a xenograft mouse model to detect the effect of circMAN1A2 on the growth of gastric cancer in vivo. We knocked down the expression of circMAN1A2 in BGC823 cells with sh-circMAN1A2 lentivirus (Fig. 6a). Compared with the control group, the downregulation of circMAN1A2 expression significantly inhibited tumour growth (Fig. 6b). Moreover, the weight and volume of the tumours in the sh-circMAN1A2 group were significantly less than that in the control group (Fig. 6c, d). These results suggest that downregulation of circMAN1A2 can inhibit the proliferation of gastric cancer cells in vivo.

### CircMAN1A2 was overexpressed in GC tissues and the preoperative plasma of GC patients

To further study the correlation between circMAN1A2 and pathological parameters in patients with GC, we detected the expression of circMAN1A2 in GC tissues. Detailed clinical pathologic information of these patients with GC was presented in Table 1. The results showed that circMAN1A2 was significantly overexpressed in GC tissues compared with adjacent tissues (Fig. 6e). In addition, the expression level of circMAN1A2 was even higher in the advanced stage of GC tissues than in the early stage, indicating that the high expression of circMAN1A2 might contribute to the progression of GC (Fig. 6f). Furthermore, we identified that the expression of circMAN1A2 was much higher in the preoperative plasma of GC patients than in healthy plasma (Fig. 6g). This finding suggested that circMAN1A2 might be used as a novel diagnostic marker for GC.

**Table 1:** Clinical pathological characteristics of gastric cancer patients.

Items	Case (*n* = 101) No. of patients (%)
Age	
<60	61 (60.4%)
≥60	40 (39.6%)
Gender	
Male	49 (48.5%)
Female	52 (51.5)
Tumour size	
<5	47 (46.5%)
≥5	54 (53.5%)
Adenocarcinoma differentiation degree	
Low	61 (60.4%)
Medium to high	40 (39.6%)
TNM stage	
I–II	24 (23.8)
III–IV	77 (76.2)

## Discussion

*H. pylori* is the main cause of chronic active gastritis and peptic ulcers, and persistent infection with *H. pylori* is closely related to gastric mucosal lymph tissue lymphoma and gastric cancer [31, 32]. Recent studies have found that circRNAs can regulate the occurrence and development of tumours, and they are expected to become a basic theoretical target for cancer prevention, diagnosis and treatment [16]. To explore the role of circRNAs in the development and progression of gastric cancer mediated by *H. pylori* infection, circRNAs in AGS gastric cancer cells infected with *H. pylori* were detected by circRNA sequencing technology. CircMAN1A2 was found to be upregulated significantly after *H. pylori* infection. Infection with *H. pylori* upregulated the expression of circMAN1A2 at the tissue and cell levels. CagA is one of the important carcinogenic virulence factors in *H. pylori* and is involved in the malignant transformation of gastric mucosa. However, the results showed that the abnormal expression of circMAN1A2 regulated by *H. pylori* was independent of CagA, and the interaction between living bacteria and cells was one of the necessary conditions to induce the aberrant expression of circMAN1A2. The specific molecular mechanism of *H. pylori* affecting circMAN1A2 expression needs further experimental exploration.

Downregulation of circMAN1A2 expression significantly inhibited the migration, invasion and proliferation of GC cells in vitro and circMAN1A2 knockdown inhibited xenograft tumour growth in vivo. Recently, circMAN1A2 was reported to be upregulated in nasopharyngeal cancer, oral cancer, thyroid cancer, ovarian cancer and lung cancer, indicating that circMAN1A2 might be a biomarker for malignant tumours [33].

Although studies have found that circRNAs have a variety of biological functions, such as miRNA sponging effects, binding with RNA binding proteins, and regulation of transcription and translation, the sponge adsorption function of circRNAs is currently the most widely and deeply studied mechanism [34–36]. For example, circSETD3 (Hsa_circ_0000567), as a microRNA-421 sponge, inhibits hepatocellular carcinoma growth [37]; meanwhile, hsa_circ_0072309 can absorb miR-492 and inhibit the proliferation and invasion of breast cancer cells [38]. RNAhybrid and miRanda analysis indicated that miR-1236-3p had a stronger binding ability to circMAN1A2. The luciferase reporter assay proved that circMAN1A2 could indeed bind to miR-1236-3p. Studies have confirmed that miR-1236-3p can inhibit the invasion and proliferation of gastric cancer cells by inhibiting the expression of MTA2 [28–30]. Subsequently, we found that MTA2 was upregulated in GC tissues and that the expression of circMAN1A2 and MTA2 was positively correlated in GC tissues. Therefore, circMAN1A2 could promote the progression of GC caused by *H*. pylori by regulating the miR-1236-3p/MTA2 axis.

To further confirm our findings, rescue experiments were performed. The results showed that the oncogenic effect of *H. pylori* in gastric cancer depended on circMAN1A2, and the antitumour effect of circMAN1A2 interference was weaken by miR-1236-3p inhibition in gastric cancer. Furthermore, the expression of circMAN1A2 was much higher in the preoperative plasma of GC patients than in healthy plasma. This finding suggested that circMAN1A2 might be used as a novel diagnostic marker for gastric cancer.

In summary, our study found that *H. pylori* could upregulate the expression of circMAN1A2 and affect the occurrence and development of gastric cancer by regulating the circMAN1A2-miR-1236-3p-MTA2 axis. *H. pylori*-circMAN1A2-miR-1236-3p-MTA2, a new signalling cascade, provides novel research ideas for clarifying the oncogenic mechanism of *H. pylori* and provides an experimental basis for exploring the molecular mechanism by which circMAN1A2 promotes the occurrence and development of gastric cancer. Our study also provides a theoretical basis for circMAN1A2 to be a diagnostic marker and intervention target for gastric cancer treatment.

## Supplementary information

Reproducibility checklist

The original image of Western blot

Primer sequences used for Real-Time PCR

## Data Availability

All data are available in this manuscript and supplementary files.
