# Supplementary figures and images for: CircMAN1A2 is upregulated by Helicobacter pylori and promotes development of gastric cancer
Source: Cell Death Dis. 2022 Apr 28;13(4):409. doi: 10.1038/s41419-022-04811-y (PMC9051101; doi:10.1038/s41419-022-04811-y)

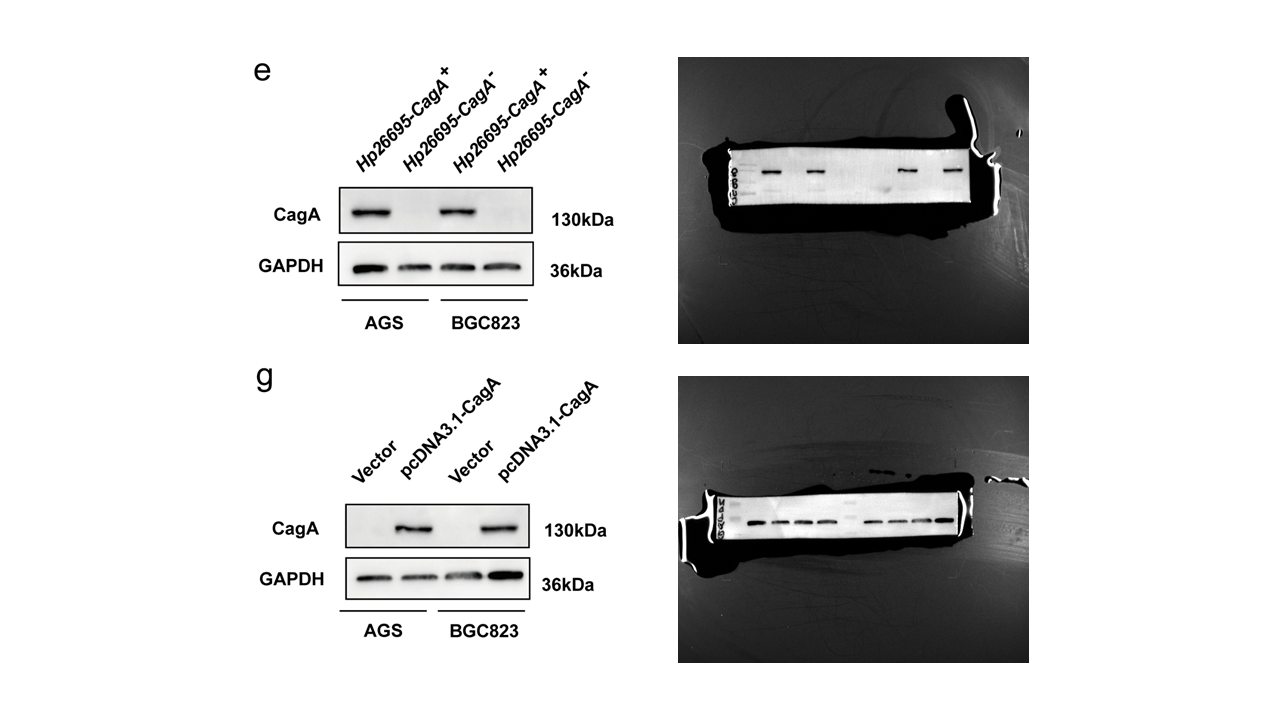

Supplement: Supplementary file 2 — The original image of Western blot [file 41419_2022_4811_MOESM2_ESM.tif]
