# Supplementary material for: CircMAN1A2 is upregulated by Helicobacter pylori and promotes development of gastric cancer
Source: Cell Death Dis. 2022 Apr 28;13(4):409. doi: 10.1038/s41419-022-04811-y (PMC9051101; doi:10.1038/s41419-022-04811-y)
Supplement: Supplementary file 3 — Primer sequences used for Real-Time PCR [file 41419_2022_4811_MOESM3_ESM.docx]

**Supplementary Table 1. Primer sequences used for Real-Time PCR**

| **Name** | **Primer sequence** |
| --- | --- |
| GAPDH Forward | 5’- GTATCGTGGAAGGACTCATGAC -3’ |
| GAPDH Reverse | 5’- ACCACCTTCTTGATGTCATCAT -3’ |
| circMAN1A2 Forward | 5’- TCCTAGATGGGCAAAGATGG -3’ |
| circMAN1A2 Reverse | 5’- GCTTCTTCCAAGGCCTTCTC -3’ |
| MAN1A2 Forward | 5’- CCTATTGGTACCTATGGCGATT -3’ |
| MAN1A2 Reverse | 5’- TCTGCTGTACATCATCATGTGT -3 |
| miR-1236-3p Forward | 5’- ATCTGAAACGCGACTCACCG -3’ |
| miR-1236-3p Reverse | 5’- GACGGAGCAAGCCCCTATTC -3 |
| MTA2 Forward | 5’- ATCATTACCAGCCACCCA -3’ |
| MTA2 Reverse | 5’- CGATTATCAGATTCTCCCTC -3 |
| U2 Forward | 5’- ATCTGAAACGCGACTCACCG -3’ |
| U2 Reverse | 5’- GACGGAGCAAGCCCCTATTC -3’ |
